# Indistinguishable pattern of amygdala and hippocampus rewiring following tone or contextual fear conditioning in C57BL/6 mice

**DOI:** 10.3389/fnbeh.2013.00156

**Published:** 2013-10-29

**Authors:** Annabella Pignataro, Silvia Middei, Antonella Borreca, Martine Ammassari-Teule

**Affiliations:** ^1^Department of Experimental Neurology, Laboratory of Psicobiology, Santa Lucia FoundationRome, Italy; ^2^Institute of Cell Biology and Neurobiology, National Research CouncilRome, Italy

**Keywords:** contextual fear conditioning, tone fear conditioning, hippocampus, amygdala, spine density, spine morphology

## Abstract

Changes in neuronal connectivity occurring upon the formation of aversive memory were examined in C57BL/6 (C57) mice 24 h after they were trained for tone fear conditioning (TFC) and contextual fear conditioning (CFC). Although TFC and CFC are amenable to distinct learning systems each involving a specific neural substrate, we found that mice trained in the two protocols showed the same increase in spine density and spine size in class I basolateral amygdala (BLA) and in dorsal hippocampus CA1 pyramidal neurons. Our findings suggest that, because of their remarkably functional hippocampus, C57 mice might engage this region in any fear situation they face. These observations raise a point relevant to aversive memory studies, i.e., how the peculiarity of memory in certain individuals impacts on the components of the fear circuitry. It is suggested that enhanced connectivity in brain regions dispensable for specific forms of learning could considerably increase the resistance of aversive memory traces to treatments aimed at disrupting them.

## Introduction

Fear conditioning (FC), the most common model of aversive memory in rodents, is rapidly induced and persists over a considerable period of time. Main characteristics include development of classical conditioning associations with emergence of non-associative hyperarousal reactions (Sauerhöfer et al., 2012) and generalization of fear to situations sharing less common features with the original one (Balogh et al., 2002; Winocur et al., 2007). Depending on the FC paradigm, the conditioned stimulus (CS) associated with an aversive unconditioned stimulus (US), generally an electric footshock, can be an explicit stimulus (e.g., tone), or the context in which the aversive experience took place.

Studies aimed at dissecting the neural basis of FC have identified a role for the basolateral amygdala (BLA) in the formation of tone and contextual fear conditioning (CFC; Phillips and LeDoux, 1994) or the latter, a role for the hippocampus, which is required for implementing the contextual representation acting as a CS (Maren et al., 1998; Anagnostaras et al., 1999). This has led to consider the two paradigms as amenable to distinct learning systems: (i) an elemental associative learning system involving the BLA but independent from the hippocampus (Phillips and LeDoux, 1992; Paré et al., 2004) and (ii) a contextual associative learning system involving by both regions (Selden et al., 1991; Phillips and LeDoux, 1992). This dichotomy, is however, challenged by data showing that manipulation of hippocampal cholinergic activity modulates tone fear conditioning (TFC) and CFC performance in opposite ways, thus pointing to hippocampal control of amygdala function in both tasks (Desmedt et al., 1998; Calandreau et al., 2006).

Traumatic experience durably impacts on neural connectivity both in humans (Liberzon and Sripada, 2008; Sripada et al., 2012) and rodents (Lamprecht et al., 2006; Restivo et al., 2009; Ostroff et al., 2012; Heinrichs et al., 2013). To shed more light on the link between formation of fear memory and intensification of connections in fear-activated neural circuits, we recently evaluated structural remodeling, i.e., spinogenesis, in principal BLA spiny neurons in C57BL/6 (C57) mice subjected to TFC. Because TFC acquisition is thought not to require the hippocampus, we also evaluated structural remodeling in dorsal CA1 pyramidal neurons as a control. Surprisingly, we found that TFC memory was associated with an increase in dendritic spines in neurons of both regions (Vetere et al., submitted).

We reasoned that this unexpected finding might be due to the specificity of the TFC protocol we used. According to this protocol (Debiec and LeDoux, 2004), that consists in exposing mice to tone-footshock pairings in context A and reactivating memory in context B, the tone-footshock pairings are delivered in two distinct contexts. Thus, in parallel with the formation of an elemental CS-US association, mice might be engaged in operations of detection/comparison of context A and B features implicating the hippocampus. Alternatively, because of their remarkably functional hippocampus (Barber et al., 1974; Matsuyama et al., 1997; Nguyen et al., 2000), C57 mice might form a hippocampus-dependent contextual representation of any fear situation they face. To clarify this point, we trained C57 mice in standard TFC and CFC paradigms, and then measured the density and size of dendritic spines in BLA and CA1 regions 24 h after each training episode. Here we show that any fear experience in this mouse strain elicits the same strong rewiring in BLA and CA1 neurons.

## Methods

### Animals

Adult 10 week old male C57BL6/J were housed in groups of four in transparent Plexiglas cages placed in a room maintained at a constant temperature (22°C) with 12/12 and light-dark cycle and food and water *ad libitum*. All experiments were carried out in accordance with the guidelines laid down by the European Communities Council Directive (86/609/European Economic Community (EEC)).

### Experimental design

The experimental design for the behavioral experiments included four conditions: CFC, pseudo-CFC conditioning (CFC-NOSHOCK), TFC, and Pseudo-TFC conditioning (TFC-NOSHOCK). In each condition, mice were subjected to one conditioning trial and one memory trial run 24 h later. All mice were handled for 5 min for three consecutive days, in the experimental room to minimize conditioning-independent emotional reactions. Mouse behavior was videotaped and fear memory was assessed by manually scoring the total amount of freezing behavior (defined as complete lack of movement, except for respiration) during memory testing. Values are reported as percentage of time spent freezing. To selectively depict conditioning-induced structural remodeling of neurons, spine density was measured in independent groups of mice trained for CFC and TFC that were sacrificed 24 h after the conditioning without running the memory test.

### Contextual fear conditioning (CFC)

On day 1, mice were placed in the conditioning chamber consisting in a transparent plastic cage (21.5 × 21.5 × 35.5 cm) with a removable grid floor made of stainless steel rods. After a 2 min habituation period, five foot-shocks (0, 7 mA, 1 s) were delivered through the grid floor at 1 min intervals. Mice were returned to their home cage 1 min after the last shock terminated. CFC memory was assessed 24 h later by placing mice for 5 min in the same chamber without any footshock delivery and recording the time spent freezing. Pseudo CFC mice were placed twice in the conditioning chamber for the same duration of the conditioning and the memory test without experiencing any footshock.

### Tone fear conditioning (TFC)

TFC was run in the same conditioning chamber as CFC (context A) that was modified during memory testing (context B).** The TFC protocol was performed as in Gogolla et al. (2009) which includes two conditioned (CSs) stimuli: a CS+ (tone) preceding the footshock, and a CS− (white noise) following the footshock. This protocol is expected to generate two independent elemental CS-US associations (tone/fooshock and white noise/no footshock) decreasing the probability of implementing a “no footshock/context” configural association. TFC training consisted in a 120 s habituation period to context A, followed by the presentation of five tone-footshock-white noise pairings. The footshock (0, 7 mA, 1 s) was delivered at the end of tone (7, 5 kHz, 30 s) presentation and was immediately followed by a white noise. The five CS+/US/CS− presentations were administered at intervals ranging from 50 to 120 s. Mice were returned to their home cage 60 s after the end of the last CS−. TFC memory was assessed 24 h later by placing mice in the experimental chamber that was modified by inserting a polygonal (basement: 21.5 × 21.5 × 22.0 × 11.5 cm; height: 35.5 cm) black box (context B). After a 120 s habituation period to the novel context, mice were exposed to three CS+ (30 s) separated by intervals of 30 s and 50 s. After a 10 s interval, they were exposed to one CS− (30 s) and then left for 90 s in the conditioning chamber (context exposure). Pseudo-TFC mice were placed twice in the conditioning chamber and exposed to the same sequence of CS+ and CS− as during the conditioning and the memory test but without any footshock was administered. The total time spent freezing as well as the proportion of time spent freezing during exposure to the CS+, the CS− and the context was recorded.

### Standardization of CFC and TFC protocols

In spite of the intrinsic peculiarities of the CFC and TFC paradigms, all the conditioned mice received the same amount of footshocks (5 × 1 s) delivered at the same intensity (0.7 mA), and were subjected to a memory test of the same duration (5 min), run at the same training-to-test interval (24 h). In both paradigms, mice were introduced into the conditioning chamber kept in darkness for 5 s before the light was switched on, during both the training and the test episode.

### Golgi Cox staining

Mice were deeply anaesthetized with chloral hydrate (500 mg/kg i.p.) and perfused transcardially with 0.9% saline solution. Brains were dissected and immediately immersed in a Golgi-Cox solution (1% potassium dichromate, 1% mercuric chloride, 0.8% potassium chromate) at room temperature for 6 days. On the seventh day, brains were transferred in a 30% sucrose solution and then sectioned with a vibratome. Coronal sections (100 μm) were collected and stained according to the method described by Gibb and Kolb (1998).

### Spine density and morphology

Spine density was measured in (i) dorsal hippocampus CA1 pyramidal neurons and (ii) amygdala class I spiny neurons which are the predominant cell type found in BLA nuclei. Neurons were identified with a light microscope (Leica DMLB) under low magnification (20X/NA 0.5). Subsequently, quantification of dendritic spines was done online under higher magnification (100X/NA 1.5) using a camera (Qimaging Qicam Fast1394) connected to the microscope. Three neurons within each hemisphere were selected for each animal. On each neuron, five 30–100 μm dendritic segments of secondary and tertiary branch orders were randomly selected for spine counts. Only protrusions with a clear connection of the head of the spine to the shaft of the dendrite were counted as spines using the Neurolucida software. Statistical comparisons were made on single neuron values obtained by averaging the number of spines counted on segments of the same neuron. Furthermore, spine head diameters were measured on previously acquired images using the ImageJ (NIH, USA) software. Spines were classified into two categories: “thin” (spine head diameter < 0.55 μm) or “large” (= 0.55 μm). All analyses were carried out by an experimenter blind to the experimental condition.

### Statistical comparisons

Group differences in freezing behavior during the memory test were estimated by means of a two-way ANOVA with conditioning paradigm (TFC, CFC), and conditioning condition (training, pseudo-training) as between-factors. For the TFC memory test, a one way ANOVA was performed to compare the amount of freezing shown during exposure to the CS+ (associative component) and to the CS− and the context (non-associative components). Differences in spine density and in the proportion of large spines were estimated by means of a three-way ANOVA with conditioning paradigm (TFC and CFC), brain region (amygdala, hippocampus), and group (naïve, pseudo-trained, trained) as between-factors.

## Results

### Behaviour

**Figure 1:**
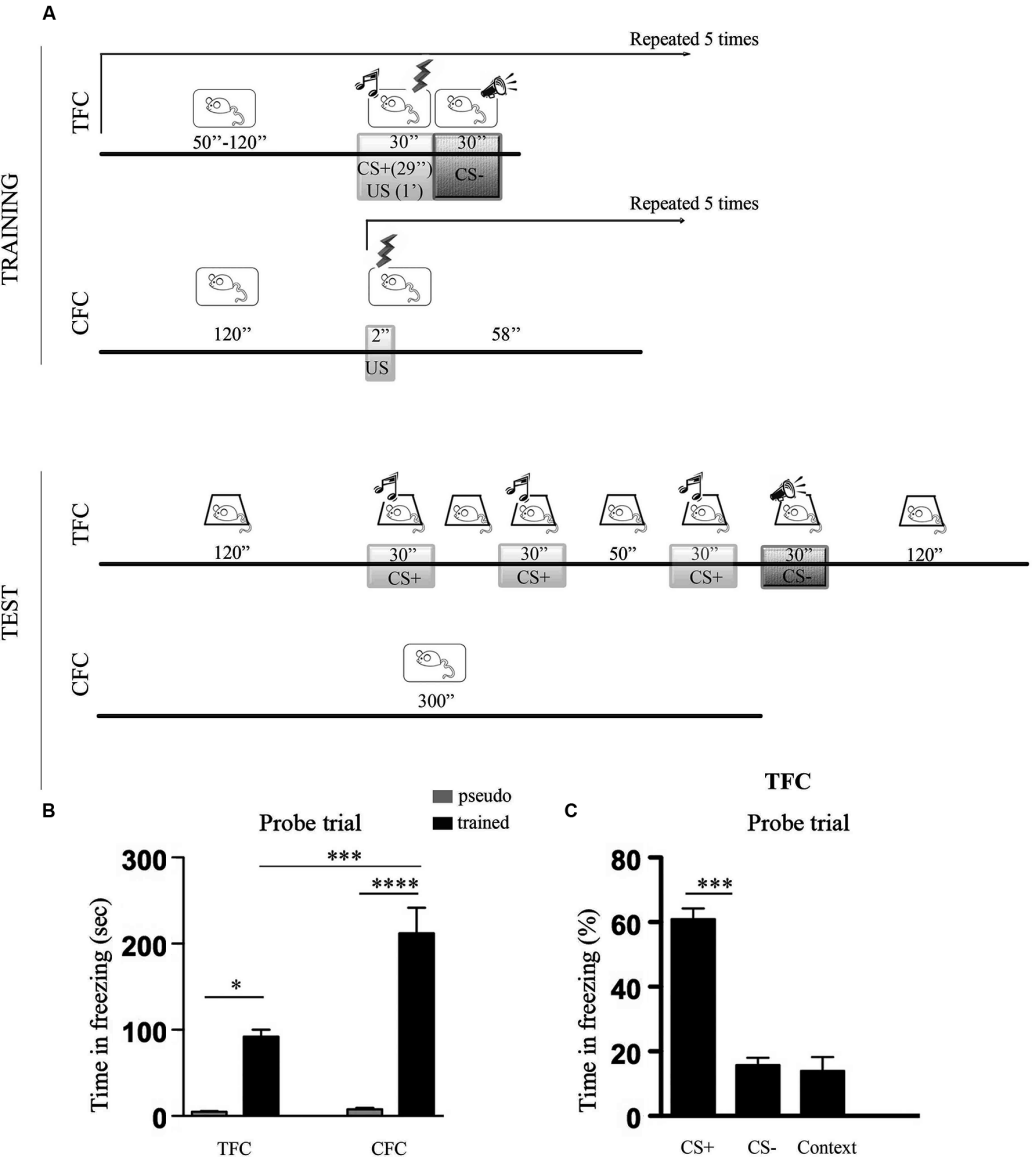
**Percentage of freezing during TFC and CFC.** **(A)** Cartoons depicting TFC and CFC training and testing protocols. ***TFC training***: after a 120 s habituation period to context A, mice were exposed to five tone-footshock pairings followed by a white noise. The tone (CS+, 7, 5 kHz, 30 s) terminated at the onset of the footshock (US, 0, 7 mA, 1 s) while the white noise (CS−) started when the footshock terminated. CS+/US/CS− presentations were administered at intervals (I) ranging from 50 s to 120 s. ***TFC testing***: mice were placed 24 h later in context B and, after a 120 s habituation period, exposed to 3 CS+ (30 s) separated by intervals of 30 s and 50 s. After a 10 s interval, they were exposed to 1 CS− and then left 90 s in the conditioning chamber (context exposure). ***CFC training***: mice were placed in context A and, after a 120 s habituation period, they were exposed to five footshocks were delivered through the grid floor at 1 min intervals. ***CFC memory testing***: mice were returned 24 h later to the same context where no footshock was delivered. **(B)** Histograms showing time spent freezing during TFC and CFC memory tests in pseudo trained (gray bars) and trained (black bars) C57BL/6 mice. TFC refers to CS+, CS− and context exposure (see Panel C for details). TFC- and CFC-trained mice showed a stronger freezing response compared to pseudo-trained mice but more freezing was recorded in the CFC than the TFC condition. **(C)** Histograms showing the percentage of freezing recorded during exposure to the associative (CS+: tone) and non-associative (CS−: white noise, context) components of the TFC memory test. Data are expressed as mean ± SEM. Mice exhibited stronger freezing during exposure to the CS+ than to the CS− or to the context, indicating that the tone was perceived as an explicit stimulus. *p < 0.05, ***p < 0.001, ****p < 0.0001.

Figure 1 shows a cartoon depicting TFC and CFC training and testing protocols (Figure 1A), histograms representing the freezing scores recorded during TFC and CFC memory tests (Figure 1B) and, for TFC, the percentage of freezing to the CS+, the CS− and to the context (Figure 1C).

The ANOVA performed on the total time spent freezing revealed a significant effect of conditioning paradigm (*F*_1,14_ = 7.34, *p* < 0.05), training condition (*F*_1,14_ = 41.37, *p* < 0.001), and of the conditioning paradigm x training condition interaction (*F*_1,14_ = 6,77, *p* < 0.05). Post hoc comparisons then indicated that all the trained mice showed significantly more freezing than the pseudo-trained mice (TFC trained *vs* pseudo-trained: *p* < 0.05; CFC trained *vs* pseudo-trained: *p* < 0.0001), but that CFC elicited stronger freezing than TFC (CFC trained *vs* TFC trained: *p* < 0.001).

Subsequent analysis of the associative *vs* non-associative components of freezing recorded during TFC memory testing revealed that mice showed significantly more freezing to the CS+ compared to the CS− and the context (*F*_2,15_ = 57,60, *p* < 0.001). The fact that mice exhibited stronger freezing during exposure to the CS+ than to the CS− or to the context points out that the tone was actually processed as an explicit CS.

### Spine density

**Figure 2:**
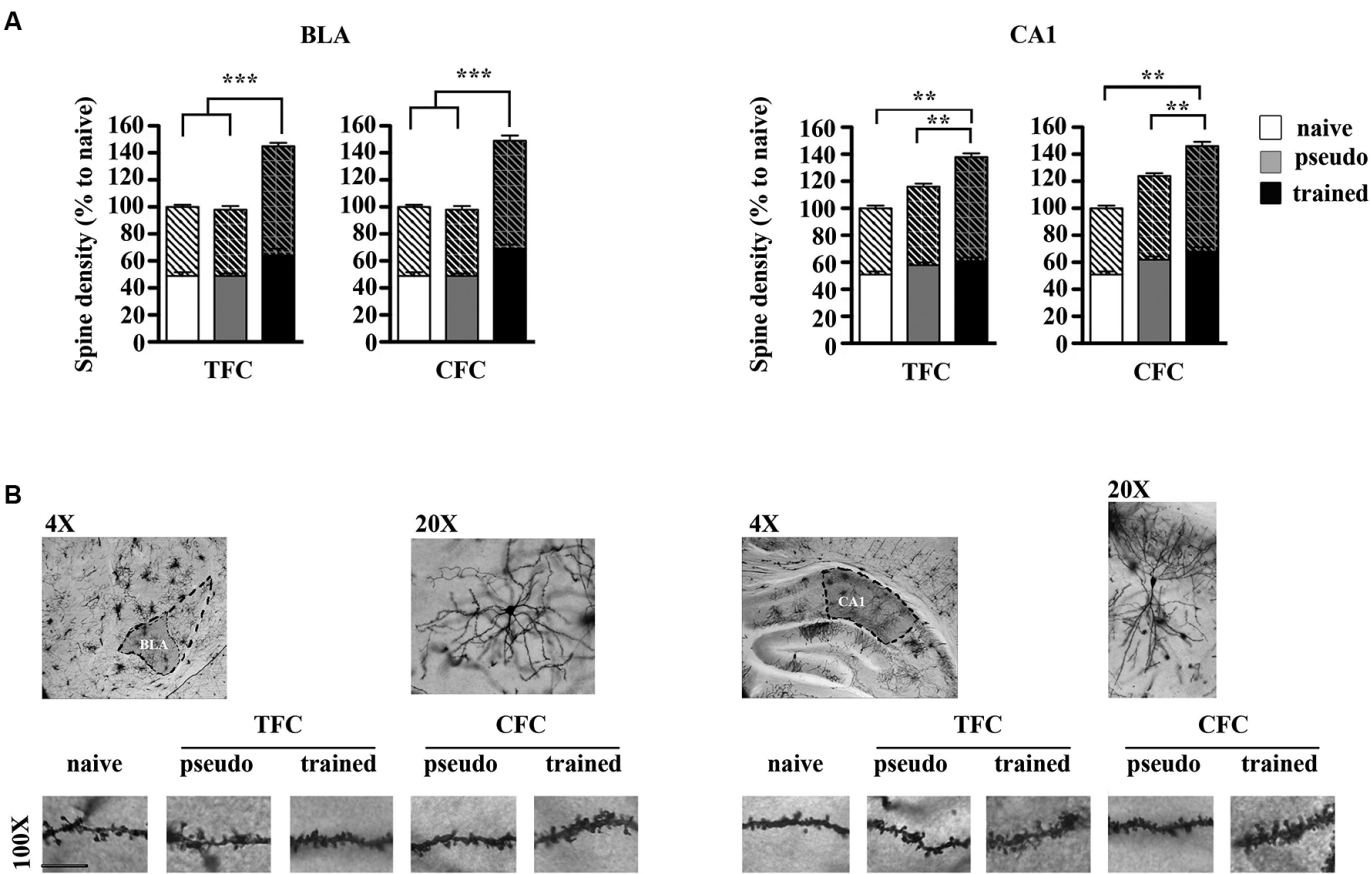
**Structural remodeling in BLA and dorsal hippocampus CA1 neurons following TFC and CFC.** **(A)** Histograms showing spine density values, with the proportion of thin (bottom) and large (top, oblique lines) spines, in class I BLA spiny neurons **(A)** and apical and basal dendrites of dorsal hippocampus CA1 pyramidal neurons **(B)** 24 h after TFC and CFC. In the BLA, spine density was increased in both TFC- and CFC-trained mice compared to pseudo-trained and naïve mice. In the hippocampus, spine density was increased in both TFC- and CFC-trained mice compared to pseudo-trained and naïve mice, but also in CFC- and TFC- pseudo-trained mice compared to naïve mice (white bars: naïve mice; gray bars: pseudo-trained mice; black bars: trained mice). **(B)** Representative segments of BLA (left) and CA1 (right) dendrite segments taken from mice experiencing each experimental condition. *p < 0.05, ***p < 0.001, ****p < 0.0001.

Figure 2 shows histograms depicting dendritic spine density values and the proportion of large spines in CA1 and class I BLA pyramidal neurons measured 24 h following TFC and CFC training. The ANOVA performed on spine density values revealed no effect of the conditioning paradigm (*F*_1,194_ = 1.88, *p* > 0.05) or of the brain region (*F*_1,194_ = 1.54, *p* > 0.05), but a significant effect of group (*F*_2,194_ = 137.53, *p* < 0.001) and group x brain region interaction (*F*_2,194_ = 6.37, *p* < 0.001). Post hoc comparisons then indicated that mice trained for CFC and TFC exhibited significantly more spines both in BLA and dorsal hippocampus CA1 neurons than their pseudo-trained and naïve counterpart (*p* < 0.001 for all comparisons) and that even CFC and TFC pseudo-trained mice exhibited more spines than the naïve mice in CA1 (*p* < 0.01 for both comparisons) but not in BLA neurons (*p* > 0.05 for both comparisons), indicating that exposure to the conditioning chamber without delivering any footshock elicits structural remodeling in the dorsal hippocampus, independently of the fact that no CS (CFC) or two CS (TFC) were delivered.

### Spine size

The ANOVA performed on large head spines values revealed no main effect of the conditioning paradigm (*F*_1,197_ = 0.24, *p* > 0.05) but an effect of group (*F*_2,197_ = 122.83, *p* < 0.001) and of the group x region interaction (*F*_2,197_ = 6.89, *p* < 0.001). Post hoc comparisons then showed that, in each region, TFC- and CFC trained mice exhibited an increased proportion of large spines relative to pseudo-trained and naïve mice (*p* < 0.01 for each comparison). In addition, the proportion of large spines was also increased in the hippocampus of TFC- and CFC- pseudo-trained mice compared to the naïve mice (*p* < 0.01). Pseudo-training did not elicit any significant change in spine size in BLA neurons (*p* > 0.05).

## Discussion

In agreement with previous reports (Paylor et al., 1994; Ammassari-Teule et al., 2000), mice showed less freezing during the TFC than during the CFC memory test. This finding can be seen as a confirmation of the low propensity of this mouse strain to form elemental stimulus-response associations independent from the context. It is, however, conceivable that this propensity becomes even stronger in a TFC protocol including two CSs: a CS+, tone, which fully predicts the onset of the shock, and a CS−, white noise, which signals that the shock is off. It is likely that delivering a CS− immediately after the US enhances the predictive value of the CS+ with the consequence of minimizing contextual generalization and non-associative hyper-arousal reactions. Supporting this view, the amount of freezing shown during exposure to the CS+ was considerably higher than during exposure to the CS− or the context.

Associative memory formation requires strengthening of synaptic connections (Gruart et al., 2006) involving phosphorylation and trafficking of glutamatergic alpha-amino-hydroxy-5,methyl-4-isoxazolepropionic acid (AMPA) receptors to synapses in hippocampus (Whitlock et al., 2006; Matsuo et al., 2008) and amygdala (Rumpel et al., 2005). Dendritic spines, which lock these molecular reorganizations in their post-synaptic densities, increase upon training in key brain regions for specific forms of learning (Restivo et al., 2009; Heinrichs et al., 2013) as the result of a higher rate of spine formation as opposed to spine elimination (Holtmaat and Svoboda, 2009; Yang et al., 2009)

Being that the amygdala is a common node of the TFC and CFC neural circuitry, we first estimated spine density in class I BLA neurons in mice trained according to each protocol but not subjected to memory testing. Measurements were performed 24 h after the training to evaluate the wiring status of BLA circuits at the time mice were otherwise tested for their memory. In agreement with recent observations (Heinrichs et al., 2013; Vetere et al., submitted) we found that spine density was significantly increased in TFC-trained mice compared to pseudo-trained and naïve mice that showed the same values. As expected from neurochemical and molecular studies showing a role of the amygdala in CFC (Desmedt et al., 1998; Calandreau et al., 2006), a similar increase in BLA spines was found following CFC training. A point to be noticed is that spines were counted on class I neurons extending throughout the BLA. Thus, although inactivation of lateral amygdala (LA) or basal amygdala (BA) regions selectively interferes with TFC and CFC (Calandreau et al., 2005), structural remodeling in the BLA was not sub-region specific.

Examination of spine size also revealed an increased proportion of large spines in both TFC- and CFC-trained mice, consistent with the view that spine enlargement provides structuralstorage sites for long-term associative memory (Bourne and Harris, 2007; Bozdagi et al., 2010). These observations therefore confirm that memorization of an aversive experience, independently of the nature of the associations supporting this memory, durably intensifies the wiring of BLA circuits.

Measurements performed in pyramidal neurons lying in the CA1 region of the dorsal hippocampus also revealed an increase in the number and the size of spines 24 h after TFC and CFC. Interestingly, a lower but significant increase in spine density was also detected in both TFC- and CFC- pseudo-conditioned mice compared to naïve mice. On the one hand, the data obtained in the CFC condition fully agree with previous observations showing that both the learning of context-shock associations as well as the introduction of mice in the conditioning chamberwithout any shock, elicit structural remodeling in dorsal hippocampus CA1 neurons (Restivo et al., 2009). On the other, the observation that TFC elicits structural changes in CA1 neurons confirms that C57 mice engage the hippocampus in a task thought to be hippocampus-independent.

An explanation for this paradoxical finding might be that, given their predisposition to form contextual representations, these mice basically implement CFC in any FC protocol. However, TFC-trained mice show more freezing to the CS+ than to the CS− or the context during the memory test, indicating that the tone was actually processed as an explicit stimulus. Alternatively, it is worth considering that, apart from its role in spatial and contextual information processing, the hippocampus is implicated in other cognitive operations, including the learning of relationships among multiple stimuli (Sutherland et al., 1989). As indicated above, we chose a TFC protocol (Gogolla et al., 2009) expected to generate two independent elemental CS-US associations (tone/footshock and white noise/no footshock) decreasing the probability of implementing a “no footshock/context” configural association. It could be, therefore, that the hippocampus was recruited for associating distinct outcomes with each CS. Nevertheless, the observation that remodeling of CA1 neurons in TFC pseudo-conditioned mice (exposed to unreinforced CS+ and CS− and to the context) was indistinguishable from the one observed in CFC-pseudo-conditioned mice (only exposed to the context) suggests that, when not followed by the footshock, the tone and the white noise are embedded in a hippocampal-based contextual representation. Consistent with this view, C57 mice need considerably more unreinforced tone presentations than other strains to show latent inhibition at the time the tone is paired with footshocks, suggesting a difficulty in disentangling the tone-alone from the context (Restivo et al., 2002).

Collectively, our results show that TFC and CFC produce indistinguishable structural changes in BLA and CA1 regions. These observations raise a point of particular relevance to aversive memory studies, i.e., how the peculiarity of memory systems in certain individuals interferes with the components of the fear circuitry. For example, it has been shown that, in humans, the genetic predisposition to build up strong memories increases the risk of developing post traumatic stress disorder (PTSD) after a traumatic event (de Quervain et al., 2012). Our data suggest that, in the same fashion, a highly functional hippocampus supporting the formation of relationships between multiple stimuli, or the inclusion of explicit stimuli in contextual/configural representations, might be recruited even in tasks where it is dispensable. This, in turn, is expected to impact on the content of fear representations, on the extension of fear-remodeled circuits, and on the resistance of aversive memory traces to treatments aimed at disrupting them (Maren, 2013).

## Author contributions

Annabella Pignataro, Silvia Middei and Martine Ammassari-Teule designed the work, Annabella Pignataro performed the experiments, Antonella Borreca achieved data analysis, Martine Ammassari-Teule wrote the manuscript.

## Conflict of interest statement

The authors declare that the research was conducted in the absence of any commercial or financial relationships that could be construed as a potential conflict of interest.
